# The importance of regional models in assessing canine cancer incidences in Switzerland

**DOI:** 10.1371/journal.pone.0195970

**Published:** 2018-04-13

**Authors:** Gianluca Boo, Stefan Leyk, Christopher Brunsdon, Ramona Graf, Andreas Pospischil, Sara Irina Fabrikant

**Affiliations:** 1 Department of Geography, University of Zurich, Zurich, Switzerland; 2 Collegium Helveticum, University of Zurich and Swiss Federal Institute of Technology in Zurich, Zurich, Switzerland; 3 Department of Geography, University of Colorado, Boulder, Colorado, United States of America; 4 National Centre for Geocomputation, University of Maynooth, Maynooth, Ireland; Colorado State University, UNITED STATES

## Abstract

Fitting canine cancer incidences through a conventional regression model assumes constant statistical relationships across the study area in estimating the model coefficients. However, it is often more realistic to consider that these relationships may vary over space. Such a condition, known as spatial non-stationarity, implies that the model coefficients need to be estimated locally. In these kinds of local models, the geographic scale, or spatial extent, employed for coefficient estimation may also have a pervasive influence. This is because important variations in the local model coefficients across geographic scales may impact the understanding of local relationships. In this study, we fitted canine cancer incidences across Swiss municipal units through multiple regional models. We computed diagnostic summaries across the different regional models, and contrasted them with the diagnostics of the conventional regression model, using value-by-alpha maps and scalograms. The results of this comparative assessment enabled us to identify variations in the goodness-of-fit and coefficient estimates. We detected spatially non-stationary relationships, in particular, for the variables related to biological risk factors. These variations in the model coefficients were more important at small geographic scales, making a case for the need to model canine cancer incidences locally in contrast to more conventional global approaches. However, we contend that prior to undertaking local modeling efforts, a deeper understanding of the effects of geographic scale is needed to better characterize and identify local model relationships.

## Introduction

Recent advances in comparative oncology have confirmed that dogs can serve as valuable models for the spontaneous development of cancer in humans [1,2]. These insights have mostly been derived from experimental studies, but spatial analyses of canine cancer can also enable the detection of risk factors for human populations, as the two species share their living environment, intimately. Such an approach to comparative oncology could be of high relevance to reducing cancer incidence in humans [3,4]. However, spatial analyses comparing canine and human cancers are currently limited, mostly because canine cancer data sources are scarce and often incomplete [5,6]. Furthermore, existing canine cancer data sources are typically compiled only within the catchment area of veterinary hospitals, thus impeding meaningful insight into risk factors for both species [7,8].

Given these data limitations, the Swiss Canine Cancer Registry (SCCR) can be considered an exceptional data source, consisting of canine cancer diagnostic records, retrospectively collected across Switzerland over a period of fifty-eight years [9,10]. Case-control studies of the SCCR have highlighted important relationships between canine cancers and a number of biological risk factors [11,12]. The same biological risk factors were also studied through spatial analyses, using conventional regression models, but the model coefficients revealed very different relationships to canine cancers [13–15]. While these results have evinced that risk factors for individuals may be difficult to detect among populations [16,17], partly as a consequence of the modifiable areal unit problem (MAUP) [18], as noted by the original authors, a number of issues still needed to be addressed in modeling canine cancer incidences [13–15].

Among these modeling issues, misspecification is especially critical in spatial analysis [19,20]. This issue can affect the estimation of the model coefficients, causing an incorrect determination of relationships between the dependent variable and the independent variables accounting for potential risk factors [21,22]. On top of this, in a conventional regression model, the coefficients are estimated “globally,” thus assuming constant relationships across all spatial units within the study area [23,24]. However, it is often more realistic to expect that the model coefficients may vary across space, because relationships are expected to change, among others, by local context. This condition, known as spatial non-stationarity, implies that the conventional regression model is inadequate [23,24], and spatial variations in the model coefficients should be computed through local models [19,25].

An essential characteristic of local models is the geographic scale, in other words, the spatial extent that is considered for estimating the local model coefficients [26,27]. One often neglected aspect is the question whether the local model coefficients depend on the geographic scale for estimation. The existence of such geographic-scale dependency could be highly problematic for the interpretation of local relationships, as there could be uncertainty as to which local coefficient better estimates the relationship of interest [26,27]. Hence, awareness of potential effects of spatial non-stationarity and geographic scale can improve the understanding of local relationships, and support a more informed interpretation of the local model coefficients. Local models enable to assess these effects by varying the bandwidth parameter [25] but have known limitations in the specification of spatial weights [28, 29].

To overcome these limitations, we designed a modeling framework inspired by the concept of regional models [30, 31]. We defined multiple regions according to a set of nearest-neighboring municipal units. Each region was identified by its central municipal unit and its geographic scale, in other words, the number of nearest-neighboring municipal units. Regional models were then fit to regions involving all possible centers and geographic scales, and selected model diagnostics were computed, summarized and visualized through value-by-alpha maps [32] and scalograms [33]. The visual representations were perused to contrast the regional models with the conventional regression model. Such a comparative assessment enabled us to uncover effects of spatial non-stationarity and geographic scale in our model of canine cancer incidences and provided elements for more informed spatial analyses of the SCCR and similar canine cancer data sources.

## Materials and methods

### Data and pre-processing

The SCCR consists of diagnostic cases collected retrospectively in Switzerland between 1955 and 2013 [9,10]. The diagnostic examinations were performed through necropsy, biopsy, and cytology tests at the reference laboratories for animal cancer diagnosis in Zurich and in Berne, as well as at a private laboratory located in the Zurich area [11,12]. Based on anonymized residential addresses (i.e., postcodes only) stored in the diagnostic data, we computed canine cancer incidences at the municipal level on a yearly basis for the period 2008–2013. For each municipal unit, the incidences were then summed over the six years. Over this period 20,209 new cancer cases were recorded in Switzerland, with a median yearly value of 3,350, and an IQR value of 127. Despite the relative stability of the yearly incidences at the country level, they vary considerably at the municipal level, with 28% of the municipal units having a median value equal or even lower than the IQR. Such a local variability justifies the aggregation of the canine cancer incidences across six years, to avoid spurious results associated with temporal variability. All types of malignant tumors were considered as cancer cases, and dogs diagnosed with more than one cancer where considered single cases.

We also accessed the Swiss canine population database, which is compiled by Animal Identity Service (ANIS) AG following the legal obligation for dog microchipping and registration established in Switzerland in 2006 [34]. Since 2008 its completeness has constantly been evaluated above 95% [12]. Using the residential address of the registered dogs, we retrieved the number of at-risk dogs at the municipal level on a yearly basis for the period 2008–2013. No exclusion criterion, as to age and sex was adopted. Similarly to the canine cancer incidences, we aggregated the population counts for each municipality over the six years, to avoid extreme fluctuations due to sample variability [21,35]. Based on the total number of incidences and the population counts recorded within municipalities over the six years, we were able to compute the average canine cancer incidence rates for the period 2008–2013.

Using the dogs registered in the Swiss canine population database, we also derived variables associated with known biological risk factors for several canine cancers [36–38] (Table 1). These variables were studied in previous spatial analyses using the SCCR data through conventional regression models [13–15]. The variables are *Average Age* (in months), *Females per Male* (in percent), and *Average Weight* (in kilograms) of the dogs registered in the different municipal units each year, during the period 2008–2013. We could not include other important biological risk factors (e.g., spaying/neutering, etc.) in this study because this information is currently not stored in the Swiss canine population database. Environmental risk factors, such as environmental tobacco smoke or air pollution in general are also not included in the current study, as these variables are difficult to obtain or impossible to compute retrospectively across Swiss municipalities for the given study years. Nevertheless, we retrieved three additional variables accounting for potential underascertainment of canine cancers (Table 1), a potential confounding factor, known to affect the study of canine cancer registry data [5,6].

**Table 1:** Median, interquartile range (IQR), minima, and maxima for the different independent variables perused in this study.

Variable	Median	IQR	Minima	Maxima
Average Age (month)	81.9	13.7	47.7	138.0
Females per Male (percent)	51.3	6.6	0.0	83.7
Average Weight (kilogram)	22.6	3.7	8.2	41.3
Dogs per Capita (percent)	13.2	8.0	1.8	276.0
Income Tax per Capita (1,000 CHF)	0.6	0.5	0.1	30.3
Distance to Veterinary Care (kilometer)	3.0	2.9	0.4	33.0

The first confounding variable refers to the urban character of municipalities. This is because lower levels of underascertainment of canine cancers are expected to occur in urban locations, where veterinary check-ups are typically more frequent [7,39]. For this purpose, we computed *Dogs per Capita* (in percent) across municipalities, using the Swiss canine population database data [34] and the Swiss Federal Statistical Office census data [40] for the period 2008–2013. This is because, different characteristics such as the status of the dog (i.e., companion versus working animal) and the type of households (i.e., smaller versus larger living spaces) in Switzerland influence the number of dogs per capita living in urban and rural municipalities [9].

Second, we considered that wealthier municipalities have reduced levels of underascertainment of canine cancers as well, because of the availability of financial means for regular veterinary check-ups [39,41]. Hence, we calculated *Income Tax per Capita* (in 1,000 Swiss Francs—CHF), by normalizing municipal income tax information collected by the Swiss Federal Tax Administration [42] and the Swiss Federal Statistical Office census data [40] for the period 2008–2012. We could not access income tax information for 2013 because the data was not publicly available at the time of the study. Despite the fact that this variable might be somehow correlated with urban status, we decided to include it separately to explore potential changes in relationships across regional models.

Third, we further addressed the frequency of regular veterinary check-ups by computing *Distance to Veterinary Care* (in kilometers) within municipal units. This was done by creating a hectometric raster (i.e., with a 100m x100m resolution) representing distances to veterinary services along roads, and averaging the raster values within those municipal units [43]. The raster was created using the addresses of the 938 veterinary services registered in the official Swiss Yellow Pages online database in 2014 [44]. The Swiss road network for 2014 was obtained as vector data from the VECTOR25 data model of the Swiss Federal Office of Topography [45]. We could not access information on the addresses of veterinary services for previous years because such historical information was not easily available to us. The projection for the raster and shapefile presented above was the Universal Transverse Mercator (UTM).

### Regression model specification and diagnostics

We fitted the average canine cancer incidence rates using a Poisson regression framework, as this is one of the most common methods for modeling disease incidences and rates of rare diseases, such as cancer [46,47]. In doing so, we relied on the assumption that the data was Poisson distributed, in particular, having the property that the conditional variance is equal to the conditional mean [48]. However, mild violations of this assumption have often been reported and accepted [49]. Given the purpose of our study, we do report the results of the over-dispersion test [50] (α = 0.05), but we did not consider alternatives to the Poisson model. This was because we focused on the systematic comparison of the model parameters and diagnostics rather than on a thorough investigation of the assumptions required for both distributions [51].

As the Poisson model is designed for modeling count data, we first fitted the observed canine cancer incidences between 2008 and 2013 (y) through the following independent variables (x)—*Average Age* (in months), *Females per Male* (in percent), *Average Weight* (in kilograms), *Dogs per Capita* (in percent), *Income Tax per Capita* (in 1,000 CHF), and *Distance to Veterinary Care* (in kilometers), according to Eq 1. The first three variables involve known biological risk factors for canine cancer, while the last three variables correct for potential underascertainment of canine cancers. The fitted canine cancer incidences were then adjusted according to the at-risk canine population between 2008 and 2013 (e), and then log-transformed, thus computing average canine cancer incidence rates for the period. In Eq 1, α is the intercept and β the multiplicative coefficient estimated for each independent variable. Note that the at-risk canine population (e) is treated differently compared to the other independent variables (x), as this is assumed to be a constant of proportionality, to allow different at-risk populations, rather than a variable used to model risk itself [46,47].

$$\log(y|x)=\alpha +\beta_{1}x_{1}+\beta_{2}x_{2\;}\ldots \;\beta_{n}x_{n}+\log\left(e\right)$$ (1)

To assess the performance of our baseline model, we perused various diagnostics about the effects (i.e., exp(ß)) resulting from the model coefficients, 95% confidence intervals (CIs), and significance levels (α = 0.05) [46,47]. The effects are interpreted as the impact of a one-unit increase in each independent variable on the expected canine cancer incidence, while the other variables are kept constant. The relative 95% CIs are also reported. When computing the significance levels and 95% CIs, we considered robust standard errors to account for possible mild deviations from the Poisson distribution [50]. We also tested the independent variables for multicollinearity to detect critical correlations among the independent variables, as this may introduce problems in the estimation of the model coefficients [52]. For this purpose, we employed the variance inflation factor (VIF) as a diagnostic and reported its square root value (SQRVIF). This is because a SQRVIF greater than 2.0 indicates a critical level of multicollinearity [53].

We then evaluated whether our baseline model provided a significant (α = 0.05) improvement over the null model, that is, the model with the intercept only. In doing so, we performed a likelihood ratio test [54] and reported the chi-squared statistic (χ^2^) [55]. To assess the goodness-of-fit, we computed the McFadden pseudo-R-squared (R^2^_McFadden_) statistic [56]. Similar to the likelihood ratio test, the R^2^_McFadden_ statistic evaluates the improvement of the baseline model over the null model with respect to the explained variability. As with the standard R-squared statistic, as a R^2^_McFadden_ statistic approaches 0, it indicates a lower model fit; a value of 1 indicates a perfect model fit [57]. In practice, the R^2^_McFadden_ statistic is more conservative, and the respective values are considerably lower than standard R-squared values. Values between 0.2 and 0.4 already suggest an excellent model fit [58].

### Spatial non-stationarity and geographic scale

In order to advance the understanding of effects of spatial non-stationarity and geographic scale, we employed the concept of regional models. This concept has been recently proposed for robust analysis and diagnostic of spatial non-stationarity and aggregation effects in epidemiologic and demographic studies [28,29]. The most important characteristic of regional models is that they keep the structure of the conventional regression model unaltered, as effects of spatial non-stationarity and geographic scale are implicitly embodied through the region to which the regression model is fit [28,29]. This results in a relatively simple modeling framework that, unlike existing local models, does not incorporate uncertainties associated with the specification of spatial weights [28, 29]. To build the regional models, we fitted the baseline model presented above within multiple regions based on a set of nearest-neighboring municipal units.

We defined the modeling regions by first considering every municipal unit as a center. Second, considering the Euclidean distance between the different centers, we iteratively selected nearest neighboring units spanning from one to the total number of municipal units within the study area [25]. These steps allowed us to define the multiple regions as a function of their centers and the number of nearest neighboring municipal units. On the one hand, this enabled us to fit models to each of the regions, thus assessing potential spatial non-stationarity in estimated relationships across regions. On the other hand, we were also able to examine the effects of geographic scale—estimated by the number of nearest neighboring municipal units involved in the regions—on these statistical relationships. However, as the geographic scale decreases, sample-size effects become critical to the regional models. For this reason, we enforced a minimum number of nearest neighboring municipal units, to ensure acceptable statistical power (β = 0.80), given a standard significance level (α = 0.05), and a small effect size (*f* ^2^ = 0.04) [59].

We contrasted the regional models using the diagnostic tools presented above, by assessing potential changes in the direction of the effects resulting from the significant model coefficients (α = 0.05) [46,47], as well as in the relative goodness-of-fit [56]. This to highlight inherent geographic variations both in the biologic risk factors and the variables accounting for the underascertainment of canine cancers. To facilitate this comparative task, we computed summary statistics for the diagnostics of the different regional models. The summary statistics were classified into quartiles to produce robust measures of central tendency (i.e., the median) and spread (i.e., the interquartile range—IQR) across the multiple diagnostics [60]. We also reported the results of the over-dispersion test (α = 0.05) for the regional models.

We then mapped the spatial distribution of both median and IQR measures for the regional models, using the location of the regions’ centers. In doing so, we built value-by-alpha maps to simultaneously depict median values through a standard continuous color scale and IQR values through variations in the alpha parameter, in other words, the opacity level [32]. This mapping technique was meant to enable a first insight into potential effects of spatial non-stationarity and geographic scale across the multiple regional models. To further investigate effects of geographic scale, we also perused scalograms, a visualization technique to assess changes in the model diagnostics across the different nearest neighboring municipal units used to define the regions [33]. On the y-axis of the filled-area plots, we present the summary statistics according to the quartile classification method, and on the x-axis, we indicate the number of nearest neighboring municipal units characterizing the regional models.

Data pre-processing, analysis, and visualization were carried out using RStudio Server v1.0.44 [61] on a Ubuntu-based computational machine (32 VCPUs and 125GB RAM), set up within the Science Cloud infrastructure of the University of Zurich, Switzerland. The following R packages were used in this study—foreach [62], gdistance [63], ggplot2 [64], maptools [65], parallel [61], plyr [66], pwr [67], reshape [68], rgdal [69], sandwich [70], and selfea [71].

## Results

### Conventional regression model

**Fig 1:**
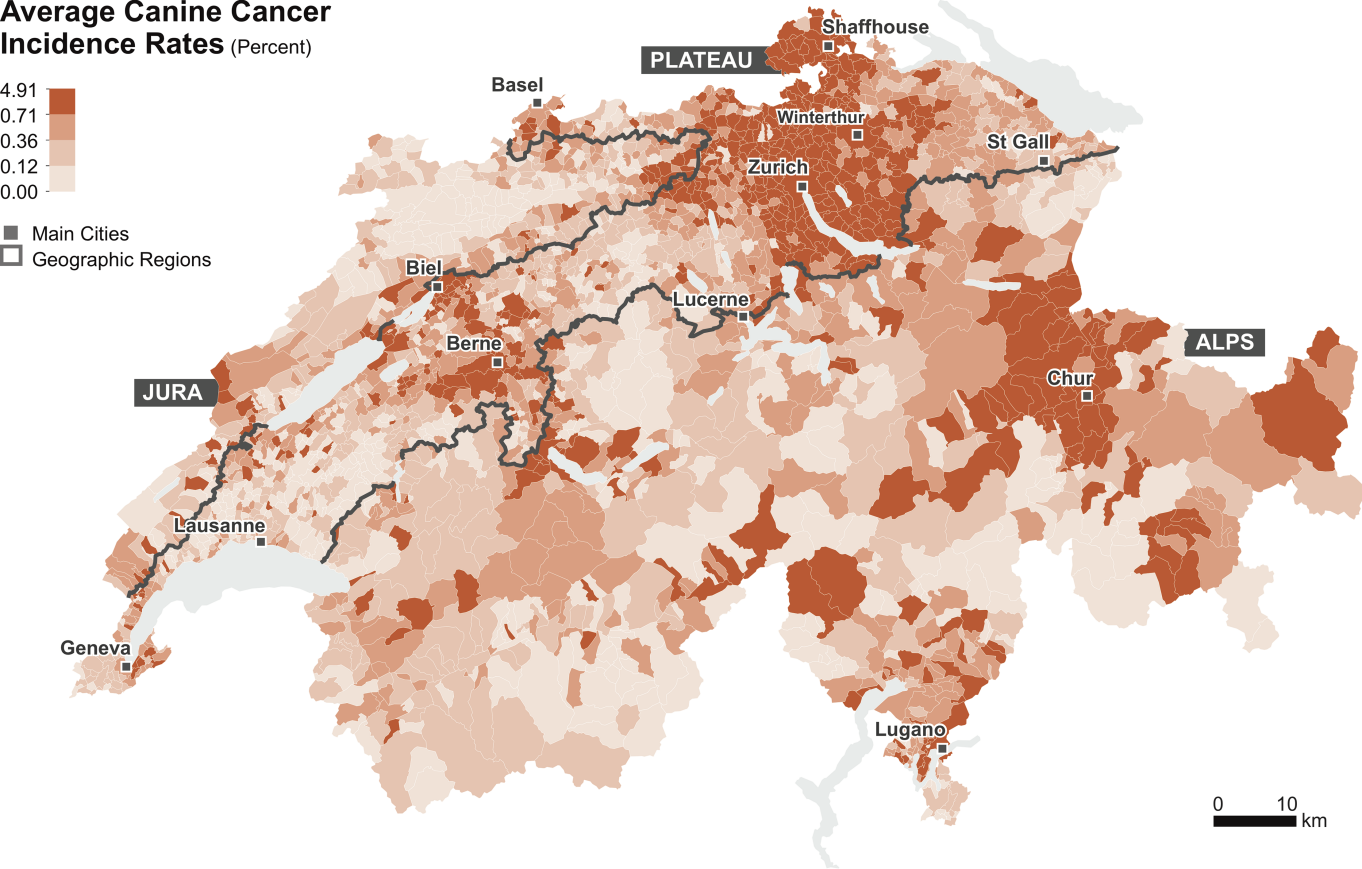
Average canine cancer incidence rates observed in Switzerland for the period 2008–2013. The data is classified according to the quantile classification.

Fig 1 shows the spatial distribution of the observed average canine cancer incidence rates for the period 2008–2013 in Switzerland, as fitted in the conventional regression model. The values are classified according to the quantile classification method to facilitate the visual interpretation. Overall, the rates ranged between 0.00% and 4.91% and presented distinct regional patterns. These patterns were dominated by higher rates in the municipal units located in the eastern part of the country, across the Cantons of Zurich and Schaffhouse (North-East), in the Canton of Grisons (East) and in the Canton Ticino (South-East). We identified additional regional patterns associated with a rural-urban cleavage. Municipal units belonging to the major urban agglomerations exhibited substantially higher rates than the rural hinterland, namely, the Cantons of Vaud, Fribourg and Berne (West), the Alps (South), and the Jura Mountain Range (North-West). Fitting the baseline model through a conventional regression model resulted in a likelihood-ratio test statistic of χ^2^ = 3,878.6 (*P* < 0.001), confirming an improvement over the model with the intercept only. Also, the R^2^_McFadden_ statistic was 0.197, suggesting a relatively good model fit. The overdispersion test returned a value of 4.3 (*P* < 0.001), indicating significant overdispersion.

Table 2 shows that all model coefficients were statistically significant (*P* < 0.05), and the SQRVIF values were consistently below 2.0, indicating the absence of critical multicollinearity. Biological risk factors, such as *Average Age* presented a negative relationship—for each increasing month, the incidences decreased by 2.0%, 95% CI [–2.4, –1.6]. Conversely, both *Females per Male* and *Average Weight* showed positive relationships—for each increasing percentage unit of female per male and each increasing kilogram, the incidences increased by 2.9%, 95% CI [2.1, 3.8] and 4.0%, 95% CI [1.9, 6.1], respectively. Confounding variables accounting for potential underascertainment of canine cancers, such as *Dogs per Capita* and *Distance to Veterinary care* exhibited negative relationships—for each increasing percentage unit of dogs and kilometer of distance, the incidences decreased by 6.0%, 95% CI [–7.2, –4.8] and 4.6%, 95% CI [–6.1, –3.1], respectively. Lastly, *Income Tax per Capita* exhibited a positive relationship—for each increasing 1,000 CHF, the incidences increased by 9.4%, 95% CI [6.1, 12.9].

**Table 2:** Effect, lower and upper 95% CI and SQRVIF for the coefficients estimated through the conventional regression model.

Coefficient	Effect	Lower CI	Upper CI	SQRVIF
Average Age (month)	0.980	0.976	0.984	1.09
Females per Male (percent)	1.029	1.021	1.038	1.03
Average Weight (kilogram)	1.040	1.019	1.061	1.22
Dogs per Capita (percent)	0.940	0.928	0.952	1.25
Income Tax per Capita (1,000 CHF)	1.094	1.061	1.129	1.03
Distance to Veterinary Care (kilometer)	0.954	0.939	0.969	1.12

### Regional models

**Fig 2:**
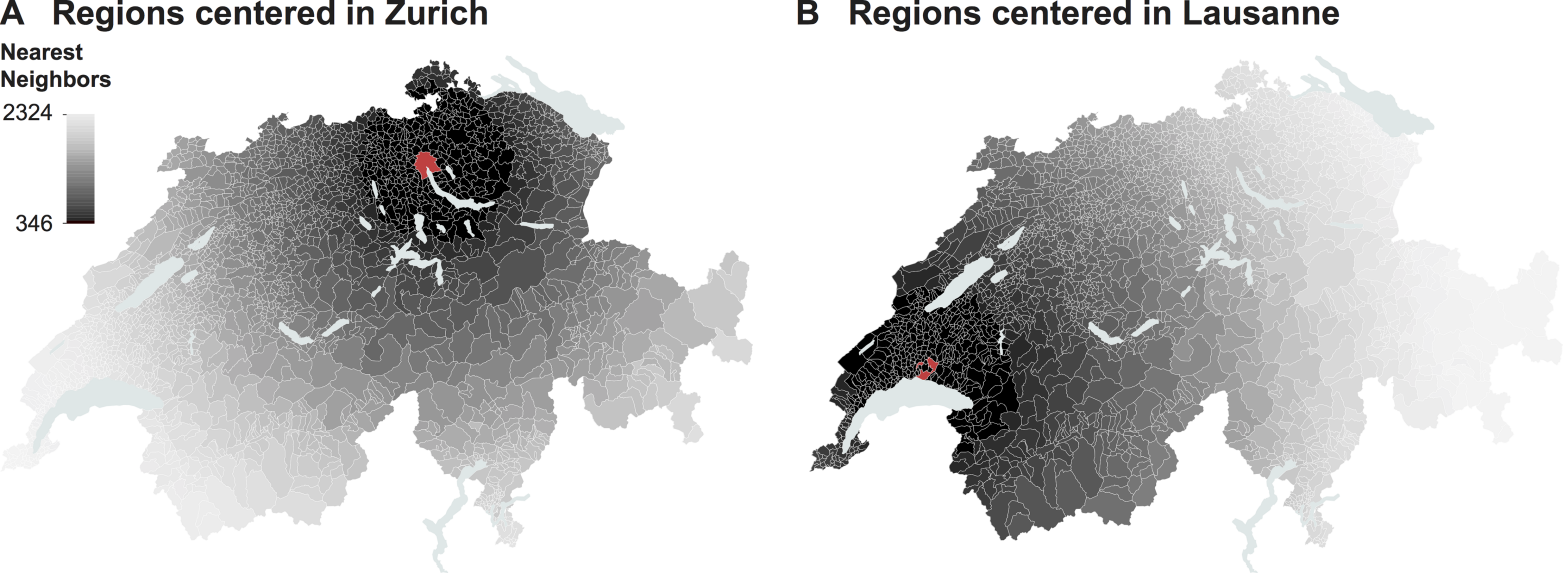
Defining regions involving different geographical scales. **Example for the regions centered in the municipality of Zurich (A) and Lausanne (B).** The center is highlighted in red.

The power analysis of the conventional regression model returned a minimum sample size of 347 municipal units. As shown in Fig 2, after excluding the center, the set of nearest-neighboring municipal units defining the multiple regions could range between 346 and 2,324. Iterating through all possible regions produced 4,594,548 regional models. In each of these models, the likelihood-ratio test statistics indicated a significant (*P* < 0.05) improvement over the model with the intercept only. The overdispersion tests returned values between 2.0 and 6.3 (*P* < 0.001), indicating significant overdispersion. None of the regional models produced model coefficients exhibiting critical multicollinearity (SQRVIF < 2.0), but, occasionally, non-significant (*P* > 0.05) model coefficients were recorded. These were discarded when producing the summary statistics and visualizations, as it is not appropriate to interpret non-significant model coefficients. Table 3 provides a first insight into the effects related to the coefficient estimated throughout the regional models.

**Table 3:** Mean, median, lower and upper 95% CI for the effects resulting from the coefficients estimated through the regional models.

Coefficient	Mean	Median	Lower CI	Upper CI
Average Age (month)	0.986	0.985	0.986	0.986
Females per Male (percent)	1.601	1.030	1.574	1.629
Average Weight (kilogram)	1.600	1.034	1.572	1.627
Dogs per Capita (percent)	1.521	0.943	1.493	1.548
Income Tax per Capita (1,000 CHF)	1.676	1.087	1.648	1.703
Distance to Veterinary Care (kilometer)	1.519	0.948	1.491	1.546

**Fig 3:**
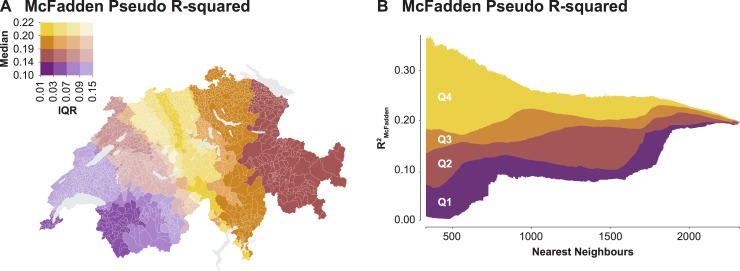
**Variations of the R**^**2**^_**McFadden**_ **measures across (A) the center and (B) the geographic scale of the regional models.** The data is classified according to the quantile classification.

Fig 3A shows the spatial variations in the R^2^_McFadden_ statistics through a value-by-alpha map. We found a clear trend in the median R^2^_McFadden_ measures, characterized by higher values in the center of the country, transitioning into lower values towards the East and the West. In the Western part of the country, we found very high IQRs, indicating a larger spread of R^2^_McFadden_ measures across geographic scales. Conversely, IQRs were closely centered around the medians in the Central and Eastern parts of the country. Fig 3B shows the variations in the R^2^_McFadden_ statistics across geographic scales using a scalogram. On the one hand, for smaller numbers of nearest neighboring units, the R^2^_McFadden_ measures exhibited a higher spread, spanning from extremely low to extremely high values. On the other hand, for larger numbers of nearest neighboring units, the R^2^_McFadden_ measures exhibited a reduced spread, becoming increasingly similar to the R^2^_McFadden_ statistic of the conventional regression model.

**Fig 4:**
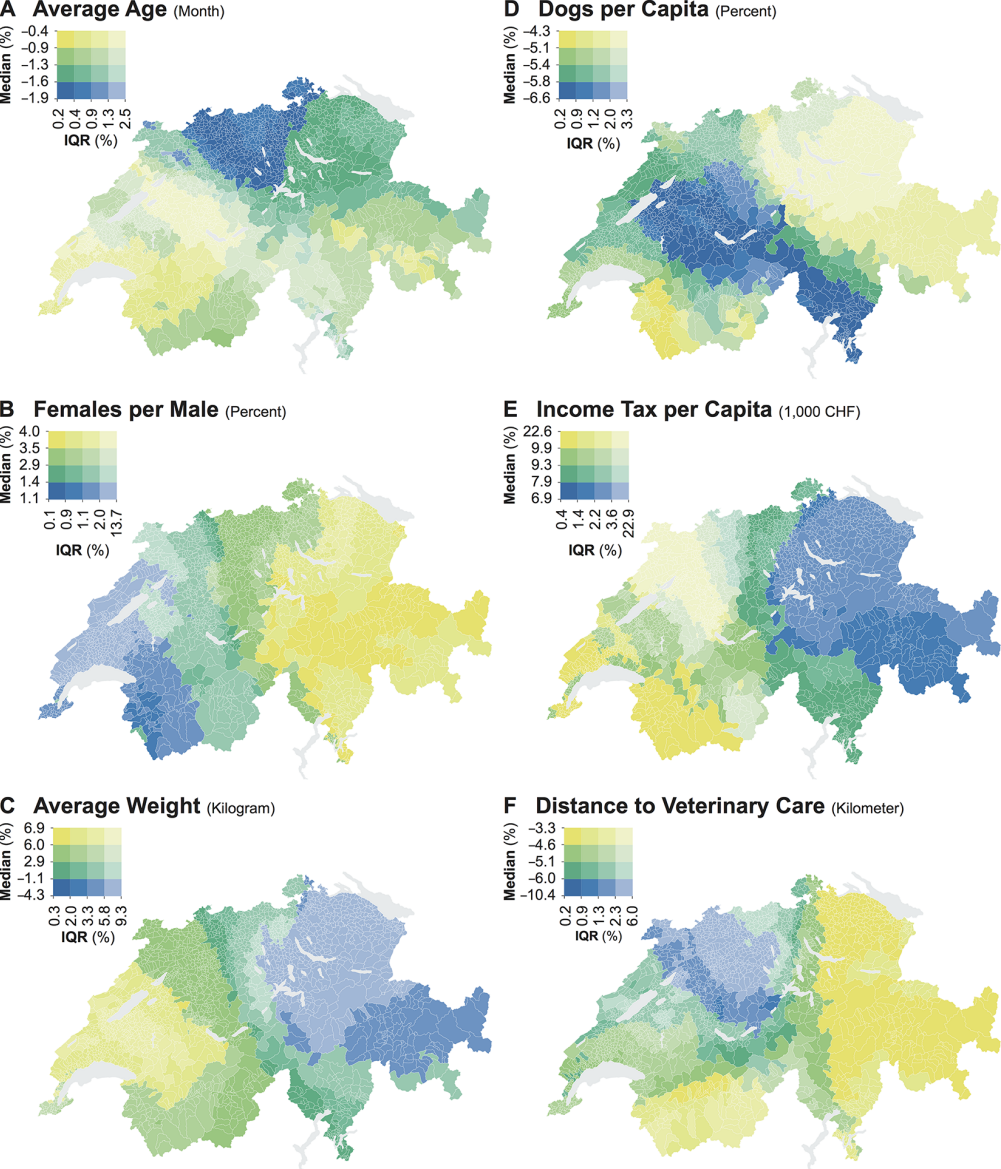
**Variations of the effects across the center of the regional models for (A) *Average Age*, (B) *Females per Male*, (C) *Average Weight*, (D) *Dogs per Capita*, (E) *Income Tax per Capita*, and (F) *Distance to Veterinary Care*.** The data is classified according to the quantile classification.

Fig 4 shows the spatial variations in the effects resulting from significant coefficient estimates through value-by-alpha maps. These revealed clear trends in the median effects, mostly across the East-West axis. In the Eastern part of the country, *Average Age* (Fig 4A) and *Average Weight* (Fig 4C), which even showed contrasting median relationships, both presented negative median effects. *Females per Male* (Fig 4B) showed positive median effects across the entire country. *Dogs per Capita* (Fig 4D) and *Distance to Veterinary Care* (Fig 4F) both showed negative median effects, while *Income Tax per Capita* (Fig 4E) presented positive median effects. All effects resulting from the significant coefficient estimates exhibited relatively high levels of spread across geographic scales, with the highest IQRs reported for *Average Weight*, *Income Tax per Capita*, and *Distance to Veterinary Care*. Nonetheless, the effects of geographic scale did not seem to follow any specific spatial distribution.

**Fig 5:**
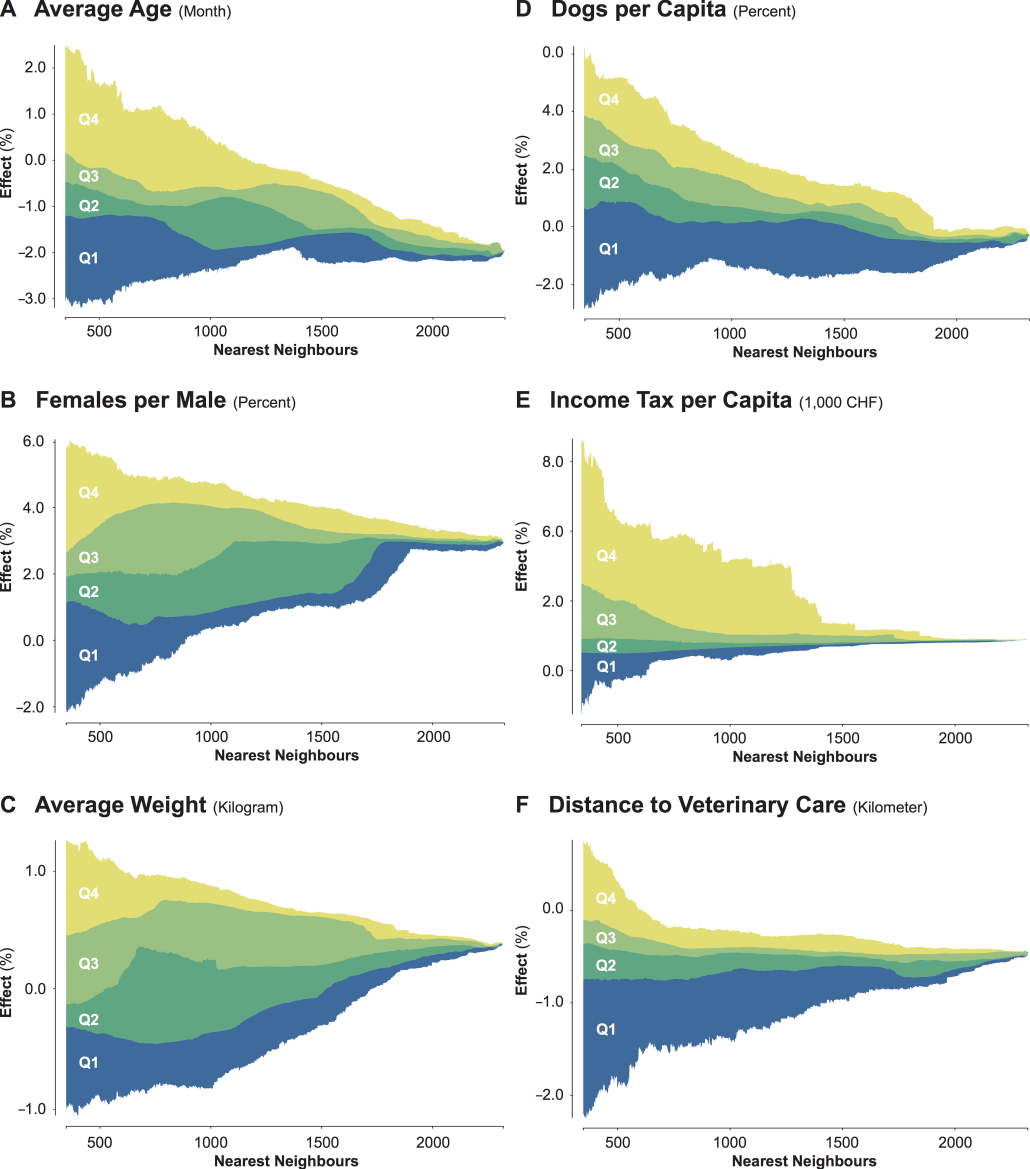
**Variations of the effects across the geographic scale of the regional models for (A) *Average Age*, (B) *Females per Male*, (C) *Average Weight*, (D) *Dogs per Capita*, (E) *Income Tax per Capita*, and (F) *Distance to Veterinary Care*.** The data is classified according to the quantile classification.

Fig 5 shows variations in the effects resulting from significant coefficient estimates across geographic scales through scalograms. These illustrate extremely high spread in the effects at smaller geographic scales, which transition into lower spreads with increasing geographic scales. *Average Age* (Fig 5A), *Females per Male* (Fig 5B), and *Average Weight* (Fig 5C) showed the highest variability of effects, which also resulted in contrasting relationships. This suggested that variables accounting for biological risk factors have both positive and negative effects, depending on the geographic scale under consideration. Conversely, the variables accounting for confounding associated with potential underascertainment of canine cancers, such as *Dogs per Capita* (Fig 5D), *Income Tax per Capita* (Fig 5E), and *Distance to Veterinary Care* (Fig 5F), showed more consistent relationships concerning geographic scale. Only sporadically did these variables exhibit both positive and negative effects, evincing important effects of geographic scale.

## Discussion

By contrasting the multiple regional models with the conventional regression model, we uncovered effects of spatial non-stationarity and geographic scale in the model of canine cancer incidences. In particular, we observed regional models with a lower goodness-of-fit, indicating regions where a finer specification of the baseline model would be necessary to reflect the relationships of interest [19,25]. These regions of poor model fit were mostly found in the rural hinterland and in the mountainous regions of the western part of the country, where lower average canine cancer incidence rates were also observed. These elements suggest that different levels of completeness of the SCCR data could be a confounder associated with potential underascertainment of canine cancers [39,41]. Further, we also identified striking effects of geographic scale, specifically over small geographic extents, where the goodness-of-fit varied greatly. These effects suggest the importance of modeling canine cancer incidences locally, in contrast to more conventional global approaches [25].

We also detected that the same model coefficient could result in contrasting effects when estimated within different regions, particularly for the variables related to biological risk factors, thus indicating spatially non-stationary relationships. On the one hand, this result could be an artifact of local selective underascertainment of canine cancers, as older dogs may be less likely to undergo regular veterinary check-ups [72]. Thus, the negative effects of *Average Age* both in the regional models and the conventional regression model. On the other hand, it is also likely that local preferences in terms of breeds could result in different effects of *Average Age* and *Average Weight* across the study area [9]. Spatially non-stationary relationships were less striking for the confounding variables accounting for potential underascertainment of canine cancers, such as *Dogs per Capita*, *Income Tax per Capita*, and *Distance to Veterinary Care*, which show more stable effects. We also reported that all relationships were affected by geographic scale to some extent, with stronger effects for *Average Age*, *Females per Male*, and *Average Weight*.

Despite these important findings, this study could have been affected by several limitations. The first set of limitations is linked to the selected modeling framework. The spatial distribution of the average canine cancer incidence rates showed clear spatial patterns, possibly violating the assumption of independence both in the regional models and the conventional regression model [73]. Also, the models were affected by over-dispersion, suggesting that the data was not perfectly Poisson-distributed [50]. Model misspecification could also be due to the non-inclusion of independent variables accounting for potential environmental exposure, such as environmental tobacco smoke [3] or air pollution [7]. The second set of limitations, which is typical of spatial analysis, is related to the assumption that the analytical units (i.e., municipal units and years) are a meaningful reflection of the relationships of interest. Aggregating individual cancer cases over municipal units for longer time spans may reduce spurious correlations due to sample variability. However, this choice is contingent on several assumptions, for instance, concerning the sedentary behavior of dogs within the municipal unit during the study period [35].

These issues will drive our future spatial analyses of canine cancer incidences. We will need to address misspecification, by including additional independent variables in the model of canine cancer incidences, and peruse a modeling framework that better accommodates the spatial (i.e., spatial autocorrelation) and statistical (i.e., overdispersion and/or zero inflation) distribution of the data—possibly through a spatially autoregressive conditional negative binomial model [74]. These measures will be implemented into the same regional modeling framework, where relationships between canine cancer incidences and both biologic risk factors and confounding factors will be assessed at different geographic scales. In doing so, we will test different machine learning methods, for instance, decision trees or clustering, to label the diagnostic measures as a function of the geographic scale [75].

## Conclusions

This study provides new insights into effects of spatial non-stationarity and scale in a model of canine cancer incidence. We fitted canine cancer incidences across Swiss municipal units through multiple regional models over a range of geographic scales. We then computed diagnostic summaries across the different spatial units and geographic scales and contrasted them with the diagnostics of the conventional regression model. The results of this comparative assessment enabled us to identify remarkable variations in the goodness-of-fit and coefficient estimates over the study area. On the one hand, this led us to speculate that misspecification and completeness in the SCCR data could be critical to our model of canine cancer incidences in some parts of the study area. On the other hand, we were able to contend that relationships were spatially non-stationary and showed geographic-scale dependency. These modeling issues were mostly detected at small geographic scales, thus making a case for the constant debate around the need to model relationships locally or regionally in contrast to more conventional regression approaches.
